# Proteomic analysis of extracellular vesicles from tick hemolymph and uptake of extracellular vesicles by salivary glands and ovary cells

**DOI:** 10.1186/s13071-023-05753-w

**Published:** 2023-04-13

**Authors:** Zhengmao Xu, Yanan Wang, Meng Sun, Yongzhi Zhou, Jie Cao, Houshuang Zhang, Xuenan Xuan, Jinlin Zhou

**Affiliations:** 1 Key Laboratory of Animal Parasitology of Ministry of Agriculture, Shanghai Veterinary Research Institute, Chinese Academy of Agricultural Sciences, Shanghai, 200241 China; 2 National Research Center for Protozoan Diseases, Obihiro University of Agriculture and Veterinary Medicine, Obihiro, Hokkaido 080-8555 Japan

**Keywords:** Extracellular vesicles, Hemolymph, Proteomics, *Rhipicephalus haemaphysaloides*, *Hyalomma asiaticum*, Ferritin-2

## Abstract

**Background:**

Extracellular vesicles (EVs) are a heterogeneous group of cell-derived membranous structures that are important mediators of intercellular communication. Arthropods transport nutrients, signaling molecules, waste and immune factors to all areas of the body via the hemolymph. Little is known about tick hemolymph EVs.

**Methods:**

Hemolymph was collected from partially fed *Rhipicephalus haemaphysaloides* and *Hyalomma asiaticum* ticks by making an incision with a sterile scalpel in the middle (between the femur and metatarsus) of the first pair of legs, which is known as leg amputation. EVs were isolated from hemolymph by differential centrifugation and characterized by transmission electron microscopy (TEM) and nanoparticle tracking analysis (NTA). Proteins extracted from the hemolymph EVs were analyzed by 4D label-free proteomics. The EVs were also examined by western blot and immuno-electron microscopy analysis. Intracellular incorporation of PHK26-labeled EVs was tested by adding labeled EVs to tick salivary glands and ovaries, followed by fluorescence microscopy.

**Results:**

In this study, 149 and 273 proteins were identified by 4D label-free proteomics in *R. haemaphysaloides* and *H. asiaticum* hemolymph EVs, respectively. TEM and NTA revealed that the sizes of the hemolymph EVs from *R. haemaphysaloides* and *H. asiaticum* were 133 and 138 nm, respectively. Kyoto Encyclopedia of Genes and Genomes and Gene Ontology enrichment analyses of identified proteins revealed pathways related to binding, catalytic and transporter activity, translation, transport and catabolism, signal transduction and cellular community. The key EV marker proteins RhCD9, RhTSG101, Rh14-3-3 and RhGAPDH were identified using proteomics and western blot. The presence of RhFerritin-2 in tick hemolymph EVs was confirmed by western blot and immuno-electron microscopy. We demonstrated that PKH26-labeled hemolymph EVs are internalized by tick salivary glands and ovary cells in vitro.

**Conclusions:**

The results suggest that tick EVs are secreted into, and circulated by, the hemolymph. EVs may play roles in the regulation of tick development, metabolism and reproduction.

**Graphical Abstract:**

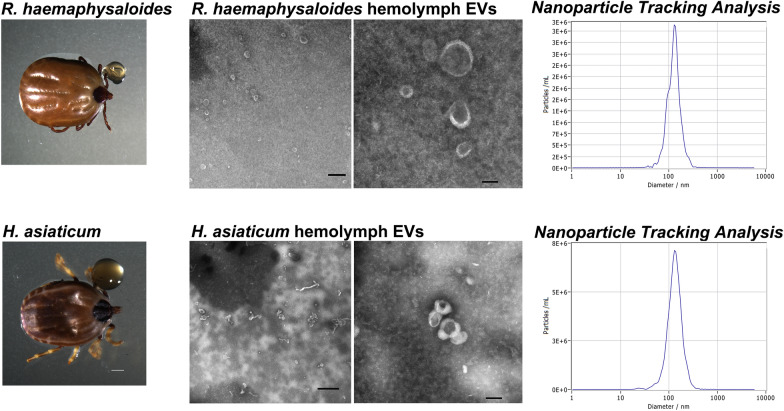

**Supplementary Information:**

The online version contains supplementary material available at 10.1186/s13071-023-05753-w.

## Background

Ticks are obligate blood-feeding ectoparasites of vertebrates and are global pests affecting human and animal health. Ticks can transmit tick-borne diseases (TBD), including protozoa, viruses, bacteria and spirochetes [1, 2], and cause substantial economic losses to the livestock industry. A better understanding of tick extracellular vesicles (EVs) could be useful for studies of tick physiology and contribute to the development of new tick control methods.

EVs are small particles enclosed by a lipid bilayer that can be released by almost any cell [3]. EVs have been found in many types of body fluids including plasma [4], cerebrospinal fluid [5], urine [6], saliva [7], tears [8], milk [9], amniotic fluid [10], seminal fluid [11] and sweat [12]. EVs incorporate various bioactive molecules from their cell of origin. These include transmembrane and cytosolic proteins, lipids and nucleic acids, which can be transferred to target cells [3]. EVs can be classified according to their size, content and mechanism of generation into exosomes (30–150 nm), microvesicles (100–1000 nm) or apoptotic bodies (50–5000 nm). They can play important role in several processes, including intercellular communication, proliferation, differentiation, angiogenesis and modulation of the immune response [13, 14]. Previous studies demonstrated that EVs are important in host-pathogen interactions, including immunomodulator presentation [15, 16], immune activation [17, 18], antiviral response [19] and infection promotion [20, 21].

EVs have been identified and characterized in many arthropod species, including ticks such as *Haemaphysalis longicornis* [22], *Ixodes scapularis* [23] and *Dermacentor andersoni* [23]. They have also been found in Diptera such as *Drosophila* [24] and *Aedes aegypti* [25], *Ornithoctonus hainana* spider [26], *Homarus americanus* lobster [27] and *Morpho* butterfly [28]. Tick saliva EVs may facilitate pathogen transmission from arthropods to mammals and participate in tick-host-pathogen relationships [22, 23].

Hemolymph in invertebrates is the equivalent of vertebrate blood. It consists of proteins, amino acids, lipids, carbohydrates, hormones, salts and cells (hemocytes) [29, 30]. The hemolymph is a circulating fluid in direct contact with all internal organs or tissues; it transports necessary substances to cells and removes metabolic waste products from the same cells [29]. The hemolymph plays an important role in the transportation of biological molecules, nutrients and hormones [31], rapid organogenesis and reorganization [32], prompting tissue development [32] and immune defense [32, 33]. The establishment of an arbovirus infection within a mosquito requires that the virus gain access to the midgut cells to multiply before disseminating into the hemolymph [34, 35]. During the initial period of rapid growth in the tick, *Borrelia burgdorferi* proliferation is restricted to the tick midgut. Upon tick engorgement, midgut resident spirochetes migrate through hemolymph to the salivary glands and are transmitted to a vertebrate host in the secreted saliva [36, 37]. EVs derived from both pathogens and their hosts have been isolated and characterized from all known pathogen classes, including viruses [38, 39], bacteria [40], fungi [41] and parasites [23, 42]. Flaviviruses use arthropod-derived EVs for viral RNA and protein transmission, which assists in viral invasion [43]. EVs purified from the hemolymph of SINV-infected *Drosophila* containing viral siRNAs confer passive protection against virus challenge in non-infected flies [24]. Given the important role of hemolymph in reproductive nutrition, signal transduction and pathogen transmission in ticks, isolation of tick hemolymph and its EVs for proteomic studies is necessary. Knowledge of hemolymph and the roles and interactions of EV proteins in ticks is scanty.

Our goal was to evaluate the EVs secreted into and circulated in tick hemolymph. We report the isolation and characterization of the hemolymph EVs from *Rhipicephalus haemaphysaloides* and *Hyalomma asiaticum* ticks. The EVs were characterized by transmission electron microscopy (TEM) and nanoparticle tracking analysis (NTA). We used 4D label-free proteomic analysis of hemolymph-derived EVs to reveal the presence of significant proteins such as GAPDH (glyceraldehyde-3-phosphate dehydrogenase), heat shock proteins, 14-3-3 (YWHA, tyrosine 3-monooxygenase/tryptophan 5-monooxygenase activation proteins) and proteases. These proteins may be used by ticks to modulate feeding, development and tick-pathogen interactions.

## Materials and methods

### Ethics approval

New Zealand white rabbits and 6–8-week-old female BALB/c mice were purchased from JSJ Laboratory Animal Co., Ltd. (Shanghai, China). Animal experiments were conducted according to the recommendations in the Guide for the Care and Use of Laboratory Animals from the Ministry of Science and Technology of the People’s Republic of China. All animal procedures were approved by the Institutional Animal Care and Use Committee of the Shanghai Veterinary Research Institute, Chinese Academy of Agriculture Sciences, P.R. China (Permit No. SV-20210702-02).

### Ticks

*Rhipicephalus haemaphysaloides* and *H. asiaticum* were maintained as previously described [44, 45]. Briefly, *R. haemaphysaloides* and *H. asiaticum* colonies were maintained in the laboratory by feeding on rabbits and incubated at 25 °C with 92% relative humidity in an incubator under complete darkness (0L:24D).

### Collection of hemolymph

For infestation, 40 *R. haemaphysaloides* or 10 *H. asiaticum* adult female ticks were attached per rabbit ear and confined using ear bags made of cotton cloth. After feeding for 6–8 days, ticks were detached/removed and hemolymph was collected as previously described [46, 47]. After washing the ticks with sterile distilled water, we immersed the ticks in 70% ethanol solution for 5 min for surface sterilization. All of the ticks were then dried using sterile paper towels and were placed to a 100-mm sterile dish. The hemolymph was collected via leg amputation. The body was then gently squeezed to produce hemolymph flow while minimizing damage to the internal organs. Hemolymph was collected with a pipette tip after an interval of 10 s.

### EV isolation from tick hemolymph and rabbit plasma

EVs were isolated from tick hemolymph by differential centrifugation, as previously described [48]. The hemolymph was mixed with an equal volume of PBS and centrifuged at 500 × *g* for 10 min at 4 °C to remove any contaminating cells. The supernatant was collected and centrifuged at 2000 × *g* for 30 min at 4 °C to remove the pallet containing dead cells. The supernatant was then collected, transferred into a new tube and centrifuged at 12,000 × *g* for 30 min at 4 ℃. The supernatant was then ultracentrifuged at 120,000 × *g* for 90 min at 4 °C in a Beckman Coulter ultracentrifuge (Optima XPN-100, Beckman Coulter Inc., Brea, CA, USA). After removing the supernatant, the hemolymph EV pellet was resuspended in sterile PBS and ultracentrifuged at 120,000 × *g* for 90 min at 4 °C. The supernatant was then removed, and the hemolymph EV was resuspended in an appropriate volume of PBS and stored at −80 °C.

Rabbit blood samples were collected into K2 EDTA tubes and then transferred to fresh tubes. The samples were centrifuged at 2000 × *g* for 30 min at 4 °C. The supernatant was collected, transferred to a new tube and then centrifuged at 12,000 × *g* for 30 min. EVs were isolated from the 12,000 × *g* supernatant by differential centrifugation, following the same protocol used for hemolymph EVs.

### Transmission Electron Microscopy (TEM)

Confirmation of EVs in the hemolymph of *R. haemaphysaloides* and *H. asiaticum* was made by TEM. The pellet was analyzed with TEM at the Shanghai Veterinary Research Institute, China. A 200-mesh copper grid with carbon-coated formvar film (Agar Scientific, Essex, UK) was incubated onto 15 μl of EV samples for 30 min. Excess liquid was removed by blotting, and grids were dried at room temperature. Grids were washed with water and stained with phosphotungstic acid for 1 min. After staining, grids were then washed with molecular-grade water. Then, the grids were examined at an acceleration voltage of 80 kV using a TEM (Tecnai G2 Spirit BIOTWIN, FEI, Eindhoven, The Netherlands).

EVs isolated from tick hemolymph were resuspended in PBS at room temperature. For immune-electron microscopy, the grids containing the EVs were incubated with 5% BSA in PBS blocking solution. Antibodies and gold conjugates were diluted in 5% BSA in PBS. After washing, the grids were exposed to the primary anti-ferritin-2 and anti-14-3-3ζ (1:100) antibody for 3 h. After washing, they were incubated for 1 h with 10 nm colloidal gold-conjugated goat anti-mouse secondary antibody (G7777, Sigma-Aldrich, St. Louis, MO, USA) diluted 1:100. After washing, the liquid remaining on the grids was blotted away, and the samples were stained with phosphotungstic acid. Grids were left to dry at room temperature and examined by TEM.

### Nanoparticle Tracking Analysis (NTA)

The NTA experiment was conducted using Zetaview PMX 120 (Particle Metrix, Meerbusch, Germany). A 1 ml sample was diluted with 1 × PBS and added to a new cell. Three cycles were performed by scanning 11 cell positions each and capturing 60 frames per position. The outliers of those measurements were removed. After capture, the videos were analyzed using ZetaView Software 8.04.02 with the following analysis parameters: maximum particle size, 1000; minimum particle size, 5; minimum particle brightness, 20. The average and median sizes and the concentration of the particles were calculated based on the data of the optimized positions.

### EV labeling

EVs were labeled with PKH26 (Red Fluorescent Cell Linker Kits MINI26; Sigma-Aldrich Co., St. Louis, MO, USA), according to the manufacturer’s protocol, with slight modifications. Briefly, tick hemolymph EVs (30 μg) and rabbit serum EVs (30 μg) were resuspended in 1 ml Diluent C, respectively. A 2 × Dye Solution was prepared in Diluent C by adding 4 μl PKH26 ethanolic dye solution to 1 ml Diluent C in a centrifuge tube and mixing well. The EV suspension was mixed with the stain solution (1:1 dilution EVs/dye) and incubated for 5 min at room temperature in a dark room. The labeling reaction was stopped by adding an equal volume of 1% BSA/fetal bovine serum. Labeled EVs were ultracentrifuged at 120,000 × *g* for 90 min, washed with PBS and ultracentrifuged again.

### EVs uptake assay

The salivary glands and ovaries were inoculated into 24-well plates (Corning Inc.) pre-added with L15C300 medium and cultured overnight at 30 °C. Then, PKH26-labeled EVs were added to the wells. After 24 h, the tissues were fixed in 4% paraformaldehyde for 30 min, washed three times with PBS and stained with Hoechst 33342 for 10 min at room temperature. After sealing with an anti-fluorescence quencher, the slides were visualized with a confocal laser-scanning microscope (Zeiss LSM 880, Germany).

### Four-dimensional label-free proteomic analysis of EVs

The 4D label-free proteomic protocol used here was described previously in [48, 49]. Briefly, SDT (4% SDS, 100 mM Tris–HCl, 1 mM DTT, pH 7.6) buffer was used for sample lysis and protein extraction. The amount of protein was quantified with a BCA Protein Assay kit (Bio-Rad, Hercules, CA, USA). Protein digestion by trypsin was performed according to a previously described filter-aided sample preparation (FASP) procedure [50]. For digestion, the protein solution was reduced with 5 mM dithiothreitol for 30 min at 56 °C and alkylated with 11 mM iodoacetamide for 30 min at room temperature in darkness. Finally, the protein suspensions were digested with 4 μg trypsin (Promega, Madison, WI, USA) in 40 μl 25 mM NH_4_HCO_3_ buffer overnight at 37 °C, and the resulting peptides were collected as a filtrate. Liquid chromatography-mass spectrometry (LC–MS/MS) analysis was performed on a timsTOF Pro mass spectrometer (Bruker Daltonics, Billerica, MA, USA) coupled to Nanoelute (Bruker Daltonics). The mass spectrometer was operated in positive ion mode. Mass spectrometric measurements were performed using the parallel accumulation serial fragmentation (PASEF) acquisition method. The MS raw data for each sample were combined and searched using MaxQuant 1.5.3.17 software for identification and quantitation analysis. The detailed methods are described in Additional file 1: Text S1.

### Gene cloning

In this study, we used an experimental approach combining sequence alignment based on tick hemolymph EV proteomics and previous *R. haemaphysaloides* transcriptome data with sequence search for the identification of tick hemolymph EV markers. The Rh14-3-3ζ, Rh14-3-3ε, RhCD9, RhTSG101, RhFerritin-1, RhFerritin-2, RhCytochrome c and RhCalnexin sequences were found in the salivary gland and midgut transcriptomes of fed *R. haemaphysaloides*. Complementary DNAs (cDNAs) were synthesized by HiScript III RT SuperMix (Vazyme, Nanjing, China) from the total RNA of the *R. haemaphysaloides* salivary gland and midgut. Gene-specific oligonucleotide primers for PCR are listed in Additional file 5: Table S1. The sequences were routinely cloned into the pMD-19 T easy vector (Takara, Dalian, China) using PrimeSTAR Max Premix (Takara), and the obtained clones were sequenced.

### Protein expression and purification

*Rhipicephalus haemaphysaloides* genes were cloned into a modified pET-30a vector containing an amino-terminal His tag. These constructs were transformed and expressed in *Escherichia coli* BL21 (DE3) strain (TIANGEN, Beijing, China). Proteins were expressed and purified, as previously described. The recombinant Rh14-3-3ζ, Rh14-3-3ε, RhFerritin-1, RhFerritin-2 and RhCalnexin proteins were purified from the soluble fraction, and the recombinant RhCD9, RhTSG101 and RhCytochrome c proteins were purified from inclusion bodies [51]. Protein expression was induced with 1 mM IPTG (isopropyl β-D-1-thiogalactoside) (Biofroxx, Einhausen, Germany) at 25 °C for 12 h. Bacteria expressing soluble proteins were lysed in Tris buffer (50 mM Tris–HCl, pH 8.0), while bacteria expressing insoluble proteins in inclusion bodies were lysed in Tris buffer containing 8 M urea. After sonication, cell lysates were centrifuged at 12,000 × *g* for 15 min, and the supernatants containing His-tagged proteins were purified by the magnetic separation using His-tag Protein Purification Magnetic Beads (Beaverbio, Suzhou, China). The beads were washed six to eight times by magnetic separation method. The protein was eluted with 200 mM imidazole and dialyzed in Tris buffer to remove imidazole and urea. The protein was loaded into a dialysis tubing (Biosharp, Anhui, China) and dialyzed for 24 h against a 2 l solution of Tris buffer, with the dialysate changed every 8 h. All purification steps were performed at 4 °C to avoid protein aggregation. The protein was centrifuged at 12,000 × *g* for 10 min, and the supernatant was stored at −20 ℃ until use. After purification, the proteins were examined with 12% ExpressPlus^™^ PAGE Gels (Genscript, Nanjing, Jiangsu, China), and the recombinant protein concentration was determined by a BCA protein assay.

### Antibody generation

The first antigen dose was a mixture of 100 μg of recombinant proteins and an equal volume of Freund’s complete adjuvant (Sigma, St. Louis, MO, USA) intraperitoneally injected into mice. Two additional doses of 50 μg of proteins in Freund’s incomplete adjuvant (Sigma) were intraperitoneally injected in mice at 14 and 28 days. At 14 days after the third immunization, sera were collected and stored at −80 °C until use.

### Western blot

Total protein was extracted from EVs stored at −80 °C using radioimmunoprecipitation assay (RIPA) lysis and extraction buffer that included a protease inhibitor cocktail (Cat. 539134, Millipore, Billerica, MA, USA). The concentration of protein was determined by the BCA method. Equal amounts of protein (30–50 µg) from each sample were separated using 12% ExpressPlus^™^ PAGE Gels (Genscript) and transferred to polyvinylidene difluoride (PVDF) membranes (EMD Millipore Corp). The membranes were blocked with 5% bovine serum albumin for 1 h at room temperature and then incubated overnight at 4 °C with the following specific primary antibodies: mouse anti-Rh14-3-3ζ (1:200), mouse anti-Rh14-3-3ε(1:200), mouse anti-RhTSG101 (1:200), mouse anti-RhCytochrome c (1:200), mouse anti-RhCalnexin (1:200), mouse anti-RhCD9 (1:200), mouse anti-RhFerritin-1 (1:200), mouse anti-RhFerritin-2 (1:200) and rabbit anti-GAPDH (1:3000, AB2020, NCM Biotech). After incubation with the corresponding goat anti-mouse or goat anti-rabbit secondary antibodies (1:5000, Thermo Fisher Scientific, Waltham, MA, USA) for 1 h at room temperature, the membranes were incubated with NcmECL Ultra Reagent (NCM Biotech Co., Ltd) and observed under a ChemiDoc MP Imaging System (Bio-Rad).

### Statistical analysis

Fisher’s exact test was used to assess the gene enrichment of three ontologies (biological processes, cell components and molecular functions) and the Kyoto Encyclopedia of Genes and Genomes (KEGG) pathway enrichment analysis.

## Results

### *Rhipicephalus haemaphysaloides* and *H. asiaticum* hemolymph contains EVs

**Fig. 1:**
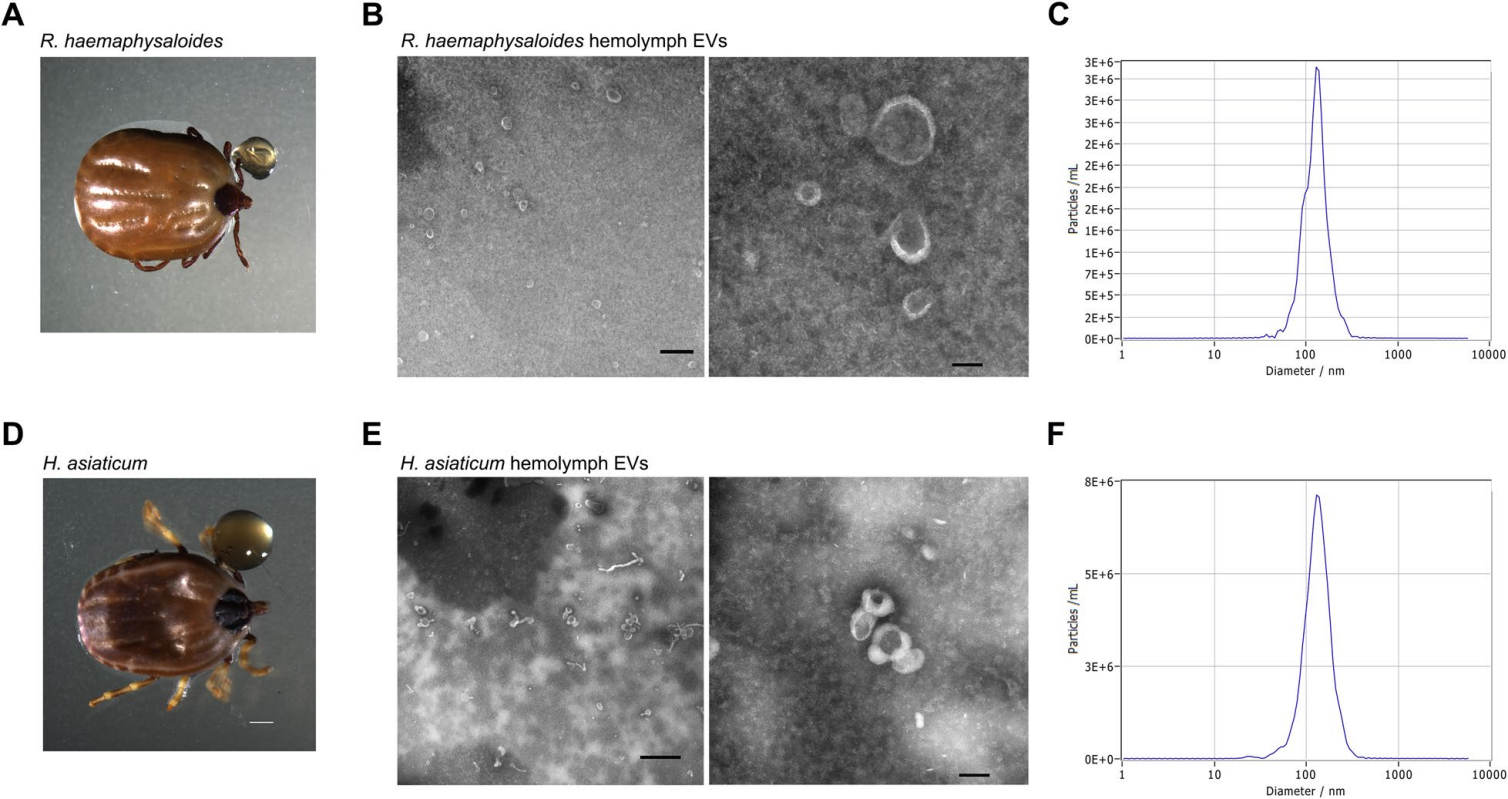
Characterization of *Rhipicephalus haemaphysaloides* and *Hyalomma asiaticum* hemolymph-derived EVs. **A**, **D** Collection of hemolymph from partially engorged *R. haemaphysaloides* and *H. asiaticum*. **B**, **E** Transmission electron micrograph of extracellular vesicles derived from tick hemolymph. Scale bar: 600 nm (left) and 100 nm (right). **C**, **F** Particle size distribution and concentrations of representative EVs were determined by nanoparticle tracking analysis of EVs (isolated from **C** *R. haemaphysaloides* and **F** *H. asiaticum* hemolymph)

Hemolymph was collected from partially fed *R. haemaphysaloides* (5–6 days post-feeding) and *H. asiaticum* (6–8 days post-feeding) ticks as shown in Fig. 1A, D. A total of 2 ml hemolymph was collected from ~ 500 *R. haemaphysaloides* ticks and ~ 300 *H. asiaticum* ticks. EVs, isolated from hemolymph, were observed by TEM and showed a typical cup-shaped morphology (Fig. 1B, E). *Rhipicephalus haemaphysaloides* hemolymph EVs (RhHEEVs) had a mean particle concentration of 5.7 × 10^9^ particles/ml and a major peak at 133.1 nm (Fig. 1C). *Hyalomma asiaticum* hemolymph EVs (HaHEEVs) had a mean size of ~ 138.3 nm and a mean particle concentration of 6 × 10^10^ particles/ml (Fig. 1F).

### Proteomic profiling of tick hemolymph EVs

The proteins in *R. haemaphysaloides* and *H. asiaticum* hemolymph-derived EVs were measured by a BCA assay. Samples containing 20 μg EV protein were resolved by SDS-PAGE (Additional file 3: Fig. S2). The SDS-PAGE gel lanes were sliced, digested with trypsin and analyzed by 4D label-free proteomic analysis. *Rhipicephalus haemaphysaloides* and *H. asiaticum* hemolymph EV protein identification, using MASCOT, confirmed the presence of 149 (Additional file 6: Table S2) and 273 proteins (Additional file 7: Table S3), respectively. Most of the proteins had been previously identified. They included heat shock proteins, transmembrane protein, histone, actin, tubulin, serine protease inhibitor, GAPDH, 14-3-3, transforming growth factor-beta-induced protein ig-h3 (TGFBI) and elongation factor 1-alpha. All of these may be intrinsic proteins of EVs (Additional file 6: Table S2, Additional file 7: Table S3).

**Fig. 2:**
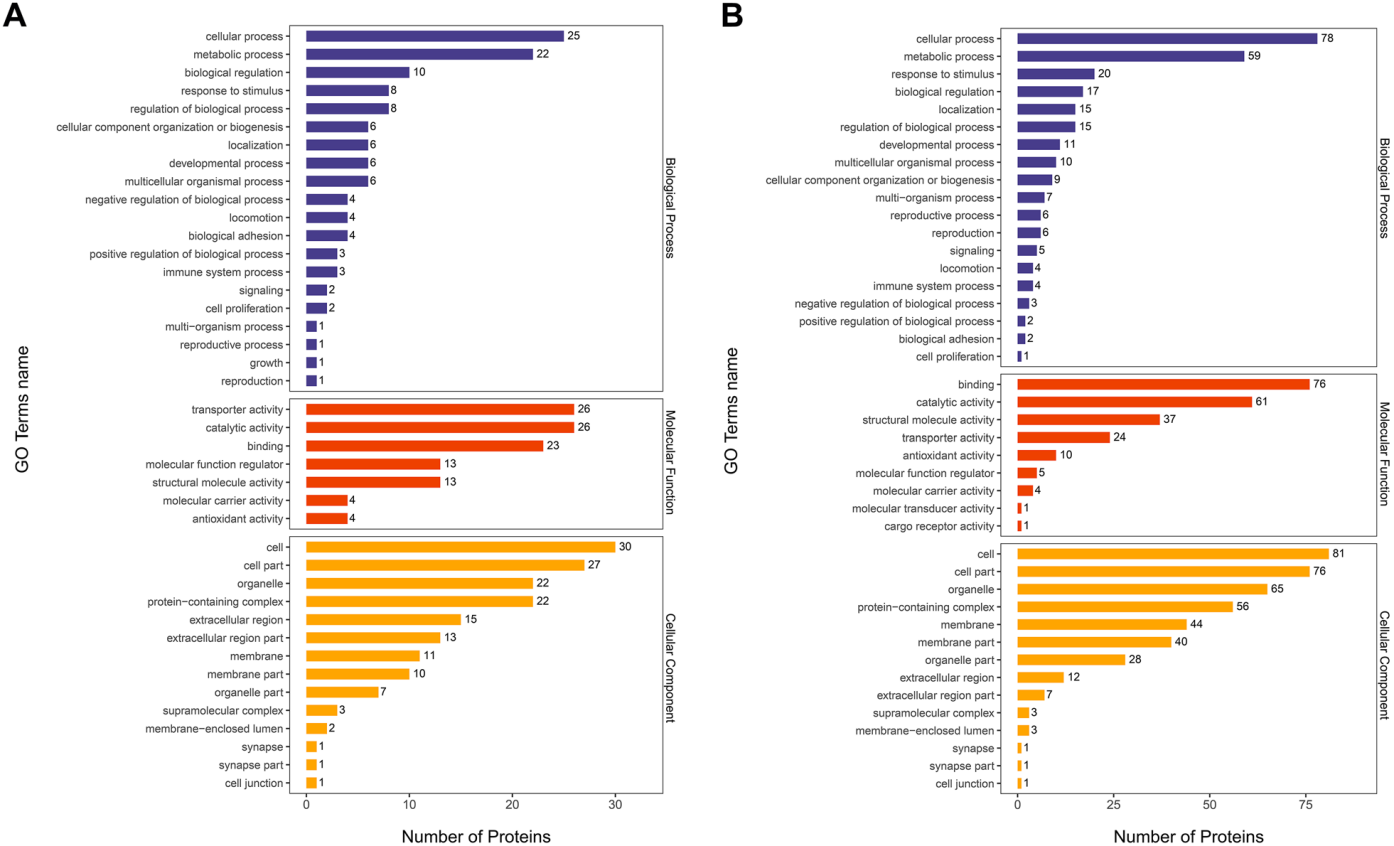
Bar graph of the Gene Ontology (GO) analysis. GO analysis of the cellular component, molecular function and biological process of the identified **A** *Rhipicephalus haemaphysaloides* and **B** *Hyalomma asiaticum* hemolymph EV proteins. The bar graph shows the distribution of corresponding GO terms. The length shows the number of all proteins associated with the GO term

To understand the biological background of the detected proteins in EVs, the Gene Ontology (GO) terms of the bioinformatic analysis tool Blast2GO were used to cluster the identified RhHEEV and HaHEEV proteins regarding their biological process, molecular function and cellular compartment (Fig. 2A, B). For biological process, most sequences are associated with cellular process (GO: 0009987; RhHEEVs: 36; HaHEEVs: 34), metabolic process (GO: 0008152; RhHEEVs: 25; HaHEEVs: 22), biological regulation (GO: 0065007; RhHEEVs: 22; HaHEEVs: 16) and response to stimulus (GO: 0050896; RhHEEVs: 16; HaHEEVs: 14) (Fig. 2A, B). For molecular function, most sequences are associated with binding (GO: 0005488; RhHEEVs: 61; HaHEEVs: 53), catalytic activity (GO: 0003824; RhHEEVs: 45; HaHEEVs: 37) and transporter activity (GO: 0005215; RhHEEVs: 37; HaHEEVs: 31) (Fig. 2A, B; Additional file 8: Table S4; Additional file 9: Table S5).

**Fig. 3:**
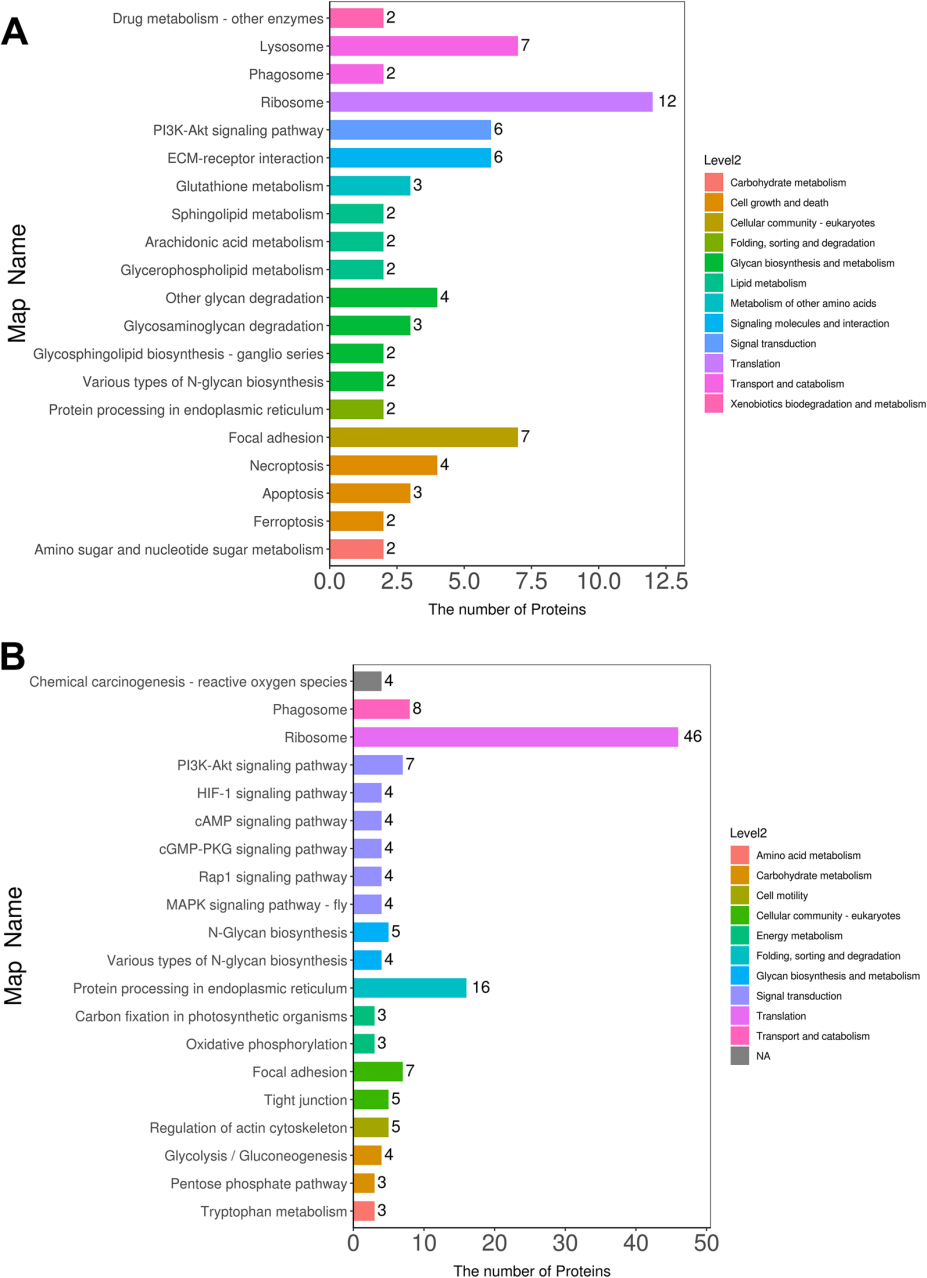
Kyoto Encyclopedia of Genes and Genomes (KEGG) pathway analysis of *Rhipicephalus haemaphysaloides* and *Hyalomma asiaticum* hemolymph EV proteome. The top 20 KEGG pathways are demonstrated in the bar diagram. The left ordinate is the name of the pathway, and the abscissa is the number of proteins related to the pathway. Different colors represent different pathways on level 2. **A** KEGG pathway enrichment column chart of *R. haemaphysaloides* hemolymph EV proteome. **B** KEGG pathway enrichment column chart of *H. asiaticum* hemolymph EV proteome

KEGG pathway analysis revealed that detected RhHEEV and HaHEEV proteins were involved in 51 and 87 pathways, respectively (Additional file 10: Table S6; Additional file 11: Table S7). The top five pathways (based on the number of proteins) in RhHEEVs were ribosome (map03010; 12), focal adhesion (map04510; 7), PI3K-Akt signaling pathway (map04151; 6), lysosome (map04142; 7) and ECM-receptor interaction (map04512; 6) (Fig. 3A; Additional file 10: Table S6,). The top five pathways (based on the number of proteins) in HaHEEVs were ribosome (map03010; 46), protein processing in endoplasmic reticulum (map04141; 16), phagosome (map04145; 8), PI3K-Akt signaling pathway (map04151; 7) and focal adhesion (map04510; 7) (Fig. 3B, Additional file 11: Table S7). These results suggest that *R. haemaphysaloides* and *H. asiaticum* hemolymph-derived EV proteins have functions in genetic information processing, regulating cellular processes and environmental information processing, especially relating to the translation, signal transduction, transport and catabolism.

### *Rhipicephalus haemaphysaloides* hemolymph and hemolymph EVs show different proteome profiles

**Fig. 4:**
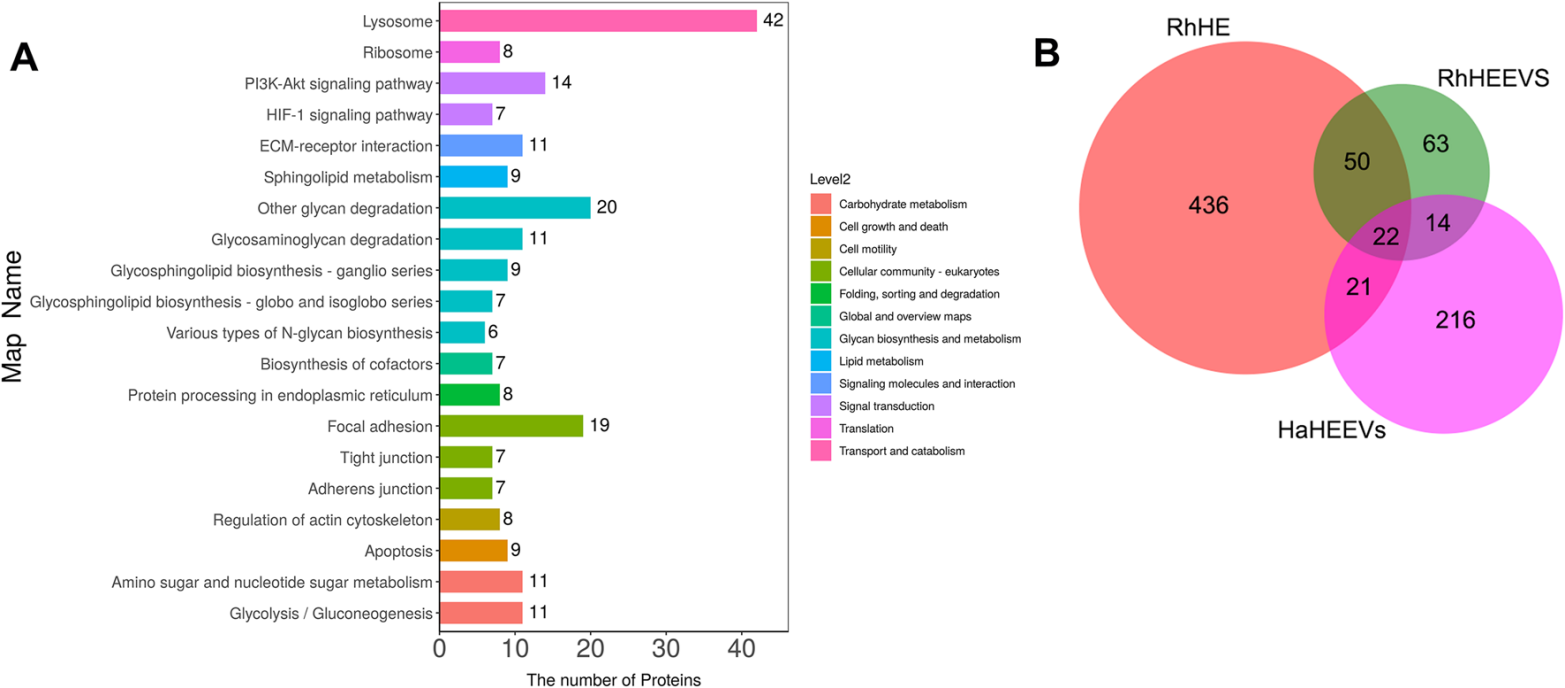
Comparison of the hemolymph and EV proteins. **A** KEGG pathway enrichment column chart of *Rhipicephalus haemaphysaloides* hemolymph proteome. **B** Venn diagram showing the number of unique and shared proteins between hemolymph and EVs

To differentiate hemolymph EV proteins with high accuracy, the 4D-label free protomic analysis was used to characterize the hemolymph proteome of *R. haemaphysaloides* females during blood feeding. A total of 529 proteins were identified (Additional file 12: Table S8). KEGG pathway analysis revealed that detected hemolymph proteins were involved in 92 pathways (Additional file 13: Table S9). The top five pathways (based on the number of proteins) were lysosome (map04142; 42), other glycan degradation (map00511, 20), focal adhesion (map04510, 19), PI3K-Akt signaling pathway (map04151, 14) and glycolysis/gluconeogenesis (map00010, 11) (Fig. 4A; Additional file 13: Table S9). We then compared the proteins in hemolymph and EVs (Fig. 4B). Among the 149 proteins identified in RhHEEVs, 77 were not detected in *R. haemaphysaloides* hemolymph (Fig. 4B). Among the 273 HaHEEV proteins, 216 were not detected in *R. haemaphysaloides* hemolymph (Fig. 4B). A total of 36 proteins were shared by RhHEEVs and HaHEEVs (Fig. 4B; Additional file 14: Table S10). These data show that > 50% of the proteins identified in the hemolymph EVs do not overlap between the two tick species. This may be due to differences in hemolymph EV abundance and variations in the gene sequences between *R. haemaphysaloides* and *H. asiaticum* (Fig. 4B).

By searching the UniProt *Oryctolagus cuniculus* database, we identified a total of 13 and 16 host proteins from RhHEEVs and HaHEEVs (Additional file 15: Table S11), respectively. Among these, alpha-globin 1, hemoglobin, albumin, alpha-2-macroglobulin and histone H2A have been previously identified in tick saliva and hemolymph proteomics [52, 53]. The presence of host-derived proteins suggests that tick hemolymph EVs may be involved in the transport of host proteins.

### Western blot characterization of hemolymph EVs

**Fig. 5:**
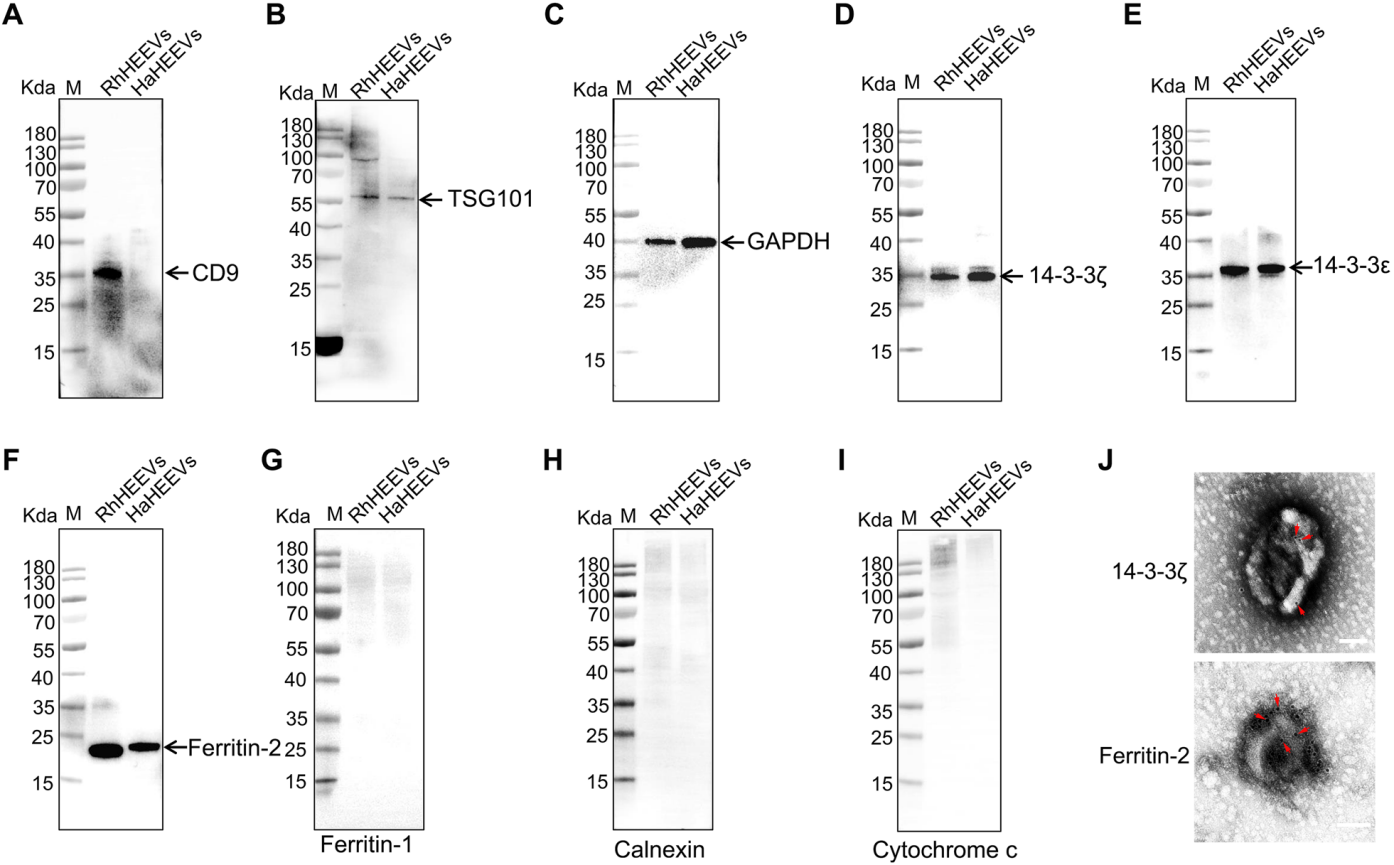
Western blot analysis of EVs purified from *Rhipicephalus haemaphysaloides* and *Hyalomma asiaticum* hemolymph. A total of 30 μg of EV protein per lane was loaded. EVs were positive for exosome markers, **A** RhCD9, **B** RhTSG101, **C** GAPDH, **D** Rh14-3-3ζ, **E** Rh14-3-3ε and **F** RhFerritin-2, and were negative for markers of **G** RhFerritin-1, **H** RhCalnexin and **I** RhCytochrome c. **J** Representative images of immunogold labeling of EVs purified by ultracentrifuge from tick hemolymph. Upper panel, 14–3-3ζ; lower panel, ferritin-2. Red arrows indicate immunogold labeling

Western blot analysis of proteomic data was conducted to further confirm the molecular composition of hemolymph EVs. For this purpose, RhCD9, RhTSG101, GAPDH, Rh14-3-3ζ, Rh14-3-3ε and RhFerritin proteins were selected. The genes encoding RhTSG101, Rh14-3-3ζ, Rh14-3-3ε, RhCytochrome c, RhCalnexin, RhFerritin-1 and RhFerritin-2 were ligated into the bacterial expression vector PET-30a, recombinants were successfully expressed as His-fusion proteins, and mouse polyclonal antibodies against them were prepared (Additional file 2: Fig. S1). Western blot analysis indicated that the RhHEEV and HaHEEV proteins were recognized by the anti-RhCD9, anti-RhTSG101, anti-GAPDH, anti-Rh14-3-3ζ, anti-Rh14-3-3ε and ani-RhFerritin-2 antibodies, but not by the anti-RhFerritin-1, anti-RhCalnexin and anti-RhCytochrome c antibodies (Fig. 5). Because RhCD9, RhTSG101, GAPDH, Rh14-3-3ζ and Rh14-3-3ε are considered representative markers of EVs, the results suggested that the isolated EVs were suitable for further analysis. RhFerritin-2 and Rh14-3-3ζ were further identified by immuno-electron microscopy on EVs (Fig. 5J). Whole-tick hemolymph lysate protein was used as the control for hemolymph cell contamination. Western blot analysis indicated that the RhHE and HaHE proteins were recognized by the anti-RhFerritin-1, anti-RhCalnexin and anti-RhCytochrome c antibodies (Additional file 4: Fig. S3). Hence, the validation of the EV proteins by western blot indicated that the proteomics data of tick-derived EVs were reliable.

### Uptake of RhHEEVs and HaHEEVs by salivary glands and ovary cells

**Fig. 6:**
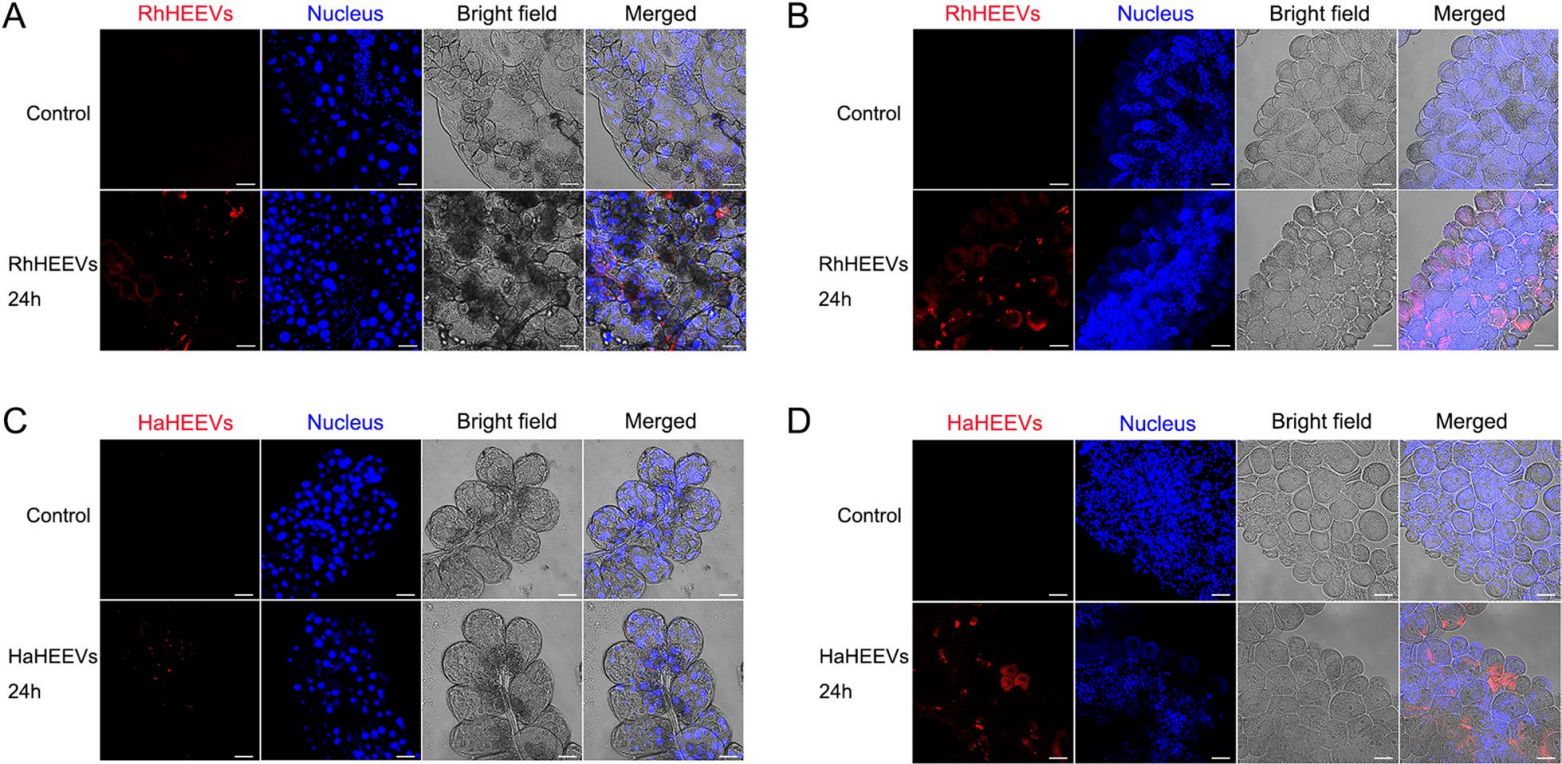
Tick organs’ uptake of PKH26-labeled hemolymph EVs. Analysis of confocal images from tick organs incubated with 10 µg/ml PKH26-stained (red) RhHEEVs or HaHEEVs and an equal volume of PBS as control, for 24 h. Cell nuclei (blue) were stained with Hoechst 33342. **A** *Rhipicephalus haemaphysaloides* and **C** *Hyalomma asiaticum* salivary glands were incubated with PKH26-stained RhHEEVs and HaHEEVs, respectively. **B** *Rhipicephalus haemaphysaloides* and **D** *H. asiaticum* ovaries were incubated with PKH26-stained RhHEEVs and HaHEEVs, respectively. Tick organs’ uptake of EVs was visualized by confocal microscopy. Scale bars, 50 μm

**Fig. 7:**
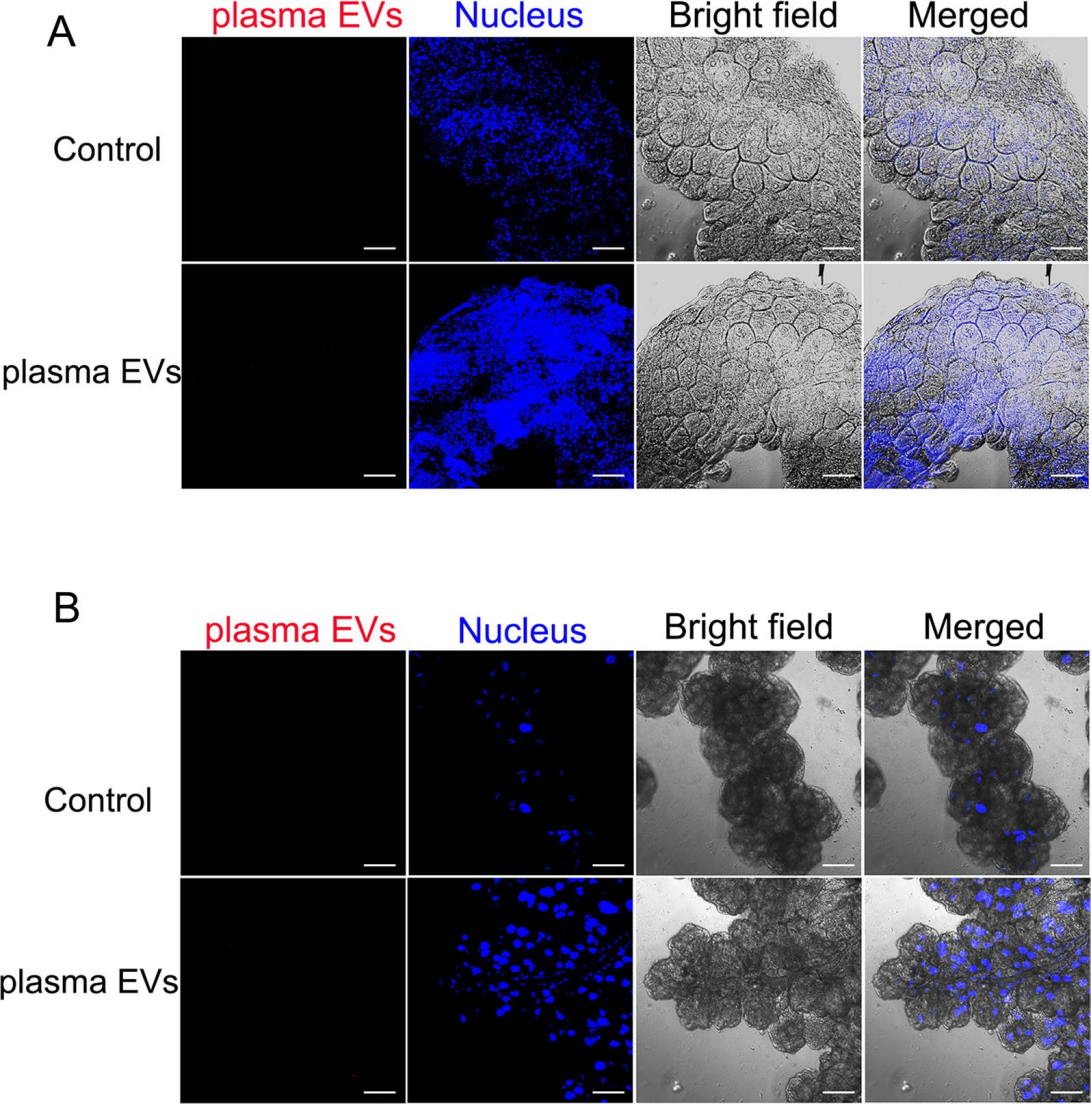
Tick organs had low uptake efficiency of PKH26-labeled rabbit plasma-derived EVs. Analysis of confocal images from tick organs incubated with 10 µg/ml PKH26-stained (red) rabbit serum EVs and an equal volume of PBS as control, for 24 h. **A** *Rhipicephalus haemaphysaloides* ovaries and **B** salivary glands were incubated with PKH26-stained rabbit plasma EVs. Tick organs’ uptake of EVs was visualized by confocal microscopy. Scale bars, 50 μm

To determine whether tick salivary glands and ovary tissues can internalize hemolymph EVs, the *Rhipicephalus haemaphysaloides* and *Hyalomma asiaticum* salivary glands and ovary tissues were incubated with PKH26-labeled RhHEEVs and HaHEEVs for 24 h, respectively. The labeled EVs were shown to enter the salivary glands and ovary tissues, and local punctate EV accumulations were observed inside the cells (Fig. 6). In contrast, the control group subjected to the same procedure did not show any intracellular fluorescence (Fig. 6A–D, upper). The EV fluorescence intensity was significantly lower in salivary gland cells than in ovary cells (Fig. 6). These results indicate that ovary tissues may absorb more EVs to regulate development. Additionally, we isolated and stained rabbit plasma EVs and tested whether they can be taken up by the tick salivary gland and ovary cells. The salivary glands and ovary tissues absorbed few if any EVs, and few local punctate EV accumulations were observed inside the cells (Fig. 7).

## Discussion

EVs have attracted research interest related to tumors, aging, diseases, pathogen transmission and being a natural drug delivery system. The significance of EVs lies in their capacity to transfer information between cells and influence functions in the recipient cells. In this study, we isolated EVs from the hemolymph of *R. haemaphysaloides* and *H. asiaticum*. TEM and NTA were used to examine EVs. Consistent with previous reports, we demonstrated that hemolymph-derived EVs are similar, in size and shape, to vertebrate and parasite-derived EVs. We also performed western blots for detecting particular proteins, such as cytochrome c and calnexin, that were not putatively expressed in EVs. CD9 was not detected in HaHEEVs by immunoblotting, which may be due to differences in the gene sequences of *R. haemaphysaloides* and *H. asiaticum*. Given that CD9, TSG101, GAPDH, 14-3-3ζ and 14-3-3ε are considered representative markers of EVs, their presence in the isolated EVs indicates that our experimental procedures provided a good yield of EVs with high purity. This facilitated downstream proteomic analysis.

In total, 149 and 273 proteins were identified in EVs isolated from *R. haemaphysaloides* and *H. asiaticum* hemolymph, respectively. Comparative studies of the hemolymph EVs of *R. haemaphysaloides* and *H. asiaticum* resulted in the identification of 113 and 237 non-overlapping proteins, respectively. This difference may be due to intrinsic interspecies variation, from differences in EV sample collection and processing. It might also result from the identification of different proteins, since precise annotation of tick genomes and protein function remains incomplete [54, 55]. The Venn diagram showed that a discrete number of specific proteins with different biological/functional profiles were present in the EVs from the two tick species. Nevertheless, the GO classification of the hemolymph EV proteome for the two tick species was similar. There were many similar GO-classified molecular functions in *R. haemaphysaloides* and *H. asiaticum* hemolymph EVs including binding, catalytic activity and transporter activity. GO term analysis showed many prominent proteins involved in cellular process, metabolic process, biological regulation, regulation of biological process and response to a stimulus. The hemolymph is in direct contact with all internal organs. It delivers necessary substances such as nutrients to the cells, and it removes metabolic waste products from the cells. The hemolymph contains hemocytes, most of which are phagocytic cells [56, 57]. Functional annotation and pathway analyses confirmed the involvement of tick hemolymph-derived EV in several processes related to translation, signal transduction, transport and catabolism. Therefore, the EVs secreted to and circulated in the hemolymph may be participating in the regulation of development, metabolism and reproduction.

We identified multiple vitellogenins (Vgs), a phospholipoglycoprotein, the precursor of vitellin (Vn). Vg is produced in the fat body, ovary and midgut in several tick species. It is then released into the hemolymph and taken up into the developing oocyte by receptor-mediated endocytosis, where the Vg may be further processed and stored [58]. Vgs are essential for egg development and oviposition. Likewise, ferritin was identified within the tick hemolymph EVs, which plays a crucial role in blood feeding and reproduction in *H. longicornis* [59], *Ornithodoros moubata* [60], *Haemaphysalis flava* [61], *Ixodes persulcatus* [62] and *I. ricinus* [63]. These proteins have been reported in *H. longicornis* saliva EVs [52], and the association of these particular proteins with tick EVs requires further confirmation. In insects, ferritin is a classically secreted protein and has a major role in systemic iron distribution [64]. In ticks, ferritin-1 is an intracellular protein that is involved in iron storage and homeostasis and is closely related to mammalian heavy-chain ferritins [60]. The secreted ferritin-2 functions as the primary transporter of non-heme iron between the tick gut and the peripheral tissues [60, 63]. Previous studies indicated that mammalian ferritin (heavy chain and light chain) can be secreted by the EV pathway [64, 65]. We confirmed the presence of secreted ferritin-2 in hemolymph EVs by western blot and immuno-electron microscopy. This indicates that ferritin-2 may transport iron to surrounding tissues and organs through the tick hemolymph EV pathway.

Laminin peptides were identified in the tick hemolymph-EV proteomics, probably by co-sedimentation with EVs. Laminins are major signaling and structural molecules of basement membranes and modulate several diverse cellular functions. These include maintaining tissue structure, adhesion and migration, differentiation and survival [66, 67]. In oral squamous cell carcinoma (OSCC) LN1-1 cell-derived EVs, laminin-332 proteins were validated as highly expressed proteins, including laminin α3, β3 and γ2 [68]. The laminin γ2-enriched EVs of OSCC cells enhance lymphangiogenesis via integrin α3-dependent uptake by lymphatic endothelial cells (LECs) [68]. In addition, laminin γ2-deficient EVs had a reduced ability to drain into lymph nodes [68]. These studies indicated that laminins play an important role in EV uptake and transport. Therefore, laminin protein may be involved in tick hemolymph EV uptake, cell differentiation and organ development. In future research, we will use in vivo and in vitro experiments to explore the effects of laminin on tick feeding, development and EV uptake.

Some other proteins in tick EVs, such as 14-3-3, heat-shock proteins, elongation factor 1-alpha, GAPDH, sodium/potassium-transporting ATPase, serine protease inhibitor and histone, have also been identified in multiple cells, parasites and biological fluid EVs. The 14-3-3 proteins are highly conserved with several hundred identified protein interaction partners that participate in various regulatory processes. These include proteins involved in growth and development [69], signal transduction [70, 71], protein transport [72, 73], cell cycle [74] and apoptosis [75]. The 14-3-3 proteins are among the most common proteins in EVs, but knowledge of the roles of 14-3-3 proteins in ticks is sparse. The 14-3-3ζ transported by the human umbilical cord mesenchymal stromal cell exosome may upregulate the autophagic level in HK-2 cells, which can prevent the injury of cisplatin [76]. Neural stem cell-derived small EVs deliver 14-3-3t, which interacts with Beclin-1 to activate autophagy [77]. The *Leishmania major* GAPDH protein is highly enriched within the EVs secreted during infection. It binds to the AU-rich 3'-UTR region of TNF-α mRNA, limiting TNF-α production [78]. This mechanism favors the infection and development of parasites in the host. In addition, the GAPDH present in EVs can capture transferrin and lactoferrin and effectively deliver these proteins into mammalian cells [79]. These findings further demonstrate that GAPDH in EVs has multiple functions. The presence of these proteins in tick hemolymph EVs suggests that the EVs in ticks are involved in multiple biological processes. Further studies on molecular function and the underlying mechanisms in the assembly and loading of EVs are needed.

In addition to the proteins described above, the tick hemolymph-derived EVs were analyzed for the presence of host proteins. The appearance of host proteins in tick hemolymph-derived EVs could be considered contamination during collection, possibly from the tick midgut. This is consistent with previous proteomic research on tick saliva [80]. However, Liu et al. found that hemolymph collected from engorged *Haemaphysalis flava* was enriched with host proteins [53]. How these host proteins are transported from the midgut to the hemolymph is unclear. In *H. longicornis* ticks, the host-derived transferrin is transferred to the ovary through the midgut and the hemolymph [81]. Host immunoglobulin G (IgG) crosses the gut wall into the hemocoel of adult *Rhipicephalus appendiculatus* female ticks when they feed on guinea pigs [82]. The short peptides produced by hydrolysis of host hemoglobin have antimicrobial activity as demonstrated in ixodid ticks [83, 84]. These studies indicate that host proteins are involved in the biological process of ticks. Their roles in tick hemolymph EVs require further investigation.

All cells, prokaryotes and eukaryotes release EVs as part of their normal physiology and during acquired abnormalities. EVs interact with, and are taken up by, cells in many ways, including receptor-mediated endocytosis [85–87], phagocytosis [88], micropinocytosis [86] or direct fusion with the plasma membrane [89]. EVs express adhesion molecules that may be associated with the adherence of EVs to cells [90, 91]. However, the cellular and molecular basis for specific targeting to acceptor cells remains unknow. EVs can be captured by recipient cells via various mechanisms dependent on or independent of clathrin [87, 89, 92]. To validate that isolated hemolymph EVs were functional, labeled EVs were incubated with tick salivary glands and ovaries, and EV internalization was visualized using confocal microscopy. Our findings suggest that hemolymph EVs were internalized by salivary glands and ovaries. This confirmed that the surface markers or receptors critical for internalization were intact in the isolated hemolymph EVs [93]. During tick blood feeding, the biggest change in organ size occurs in the midgut, and the in vitro cultured midgut is easily damaged. Therefore, future studies need to focus on the uptake of EVs by tick organs and cells in vivo using new observation methods. Remarkably, the tick EVs identified in the hemolymph may have originated from virtually any tick cell. Therefore, it is still unclear which cells, tissues and organs the tick hemolymph EVs are released from. Interestingly, tick ovaries take up more hemolymph EVs than salivary glands. However, the mechanisms of hemolymph EV uptake by tick recipient cells or organs remain unknown.

As EVs could be released in tick hemolymph, the characterization of EVs isolated directly from hemolymph will provide useful new information. It is difficult to isolate EVs from a limited quantity of tick hemolymph. It is also a challenge to collect, handle and prepare sufficient hemolymph EVs for characterization, identification, functional analysis and tick-host-pathogen relation studies. Our research advanced the processes for the isolation and characterization of the EVs from tick hemolymph. However, the biogenesis pathways of EVs in ticks remain unexplored. We believe that tick EV transported antigens are ideal candidates for the development of transmission-blocking vaccines [94]. The proteomic profile of tick hemolymph-derived EVs is needed for developing novel vaccines or therapeutics against ticks based on EVs. Analyzing the protein composition of EVs is also critical for understanding the mechanism of their biogenesis and their functional roles in TBD transmission. Future research directions should focus on the identification of targets that could be used for the development of preventive and therapeutic methods for tick control.

Tick hemolymph EVs have the potential to provide important biological information regarding tick feeding status and tissue/organ development. However, the identification and characterization of hemolymph EV proteins that are important for tick and TBD prevention and control remain challenging and complex.

## Conclusions

The present study is the first to analyze tick hemolymph EVs. Tick EVs are secreted into, and circulated by, the hemolymph. Hemolymph EVs may play roles in the regulation of tick development, metabolism and reproduction. Ferritin-2 may act as part of the hemolymph EVs for iron transport and help regulate development, metabolism and reproduction. Our findings enhance our understanding of tick hemolymph EVs. The presence of these proteins in tick hemolymph EVs suggests that they are involved in many tick biological processes. Further studies of the molecular function of EVs and the underlying mechanisms in the assembly and loading of EVs are needed.

## Supplementary Information

**Additional file 1: Text S1.** The 4D label-free experimental procedures.

**Additional file 2: Figure S1.** Expression and affinity purification of recombinant proteins. Recombinant proteins were purified by Ni-NTA affinity column (detected by western blot). The gel was stained with Coomassie brilliant blue G250 (CBB G250). **(A)** Western blot analyses of the recombinant Rh14-3-3ζ and Rh14-3-3ε protein. The (a) panel is a gel, the (b) panel is a western blot for anti-Rh14-3-3ζ, the (c) panel is a western blot for anti-Rh14-3-3ε, the (d) panel is a western blot of a 6*His-tagged fusion protein with anti-6*His tag and the (e) panel is a western blot for anti-preserum. Lane 1, recombinant Rh14-3-3ζ protein; Lane 2, recombinant Rh14-3-3ε protein. Western blot analyses of the recombinant **(B)** RhFerritin-1, **(C)** RhFerritin-2, **(D)** RhTSG101, **(E)** RhCalnexin and **(F)** RhCytochrome c protein. For (B)–(F), all of the (a) panels are gel, the (b) panels are western blots for anti-corresponding protein, the (c) panels are western blots of the 6*His-tagged fusion protein with anti-6*His tag and the (d) panels are western blots for anti-preserum.

**Additional file 3: Figure S2.** SDS-PAGE analysis of proteins from RhHEEVs and HaHEEVs.

**Additional file 4: Figure S3.** Western blot analysis of whole *Rhipicephalus haemaphysaloides* and *Hyalomma asiaticum* hemolymph lysates. A 50 μg amount of whole hemolymph lysate protein per lane was loaded. Western blot analyses of the RhHE and HaHE proteins by **(A)** RhFerritin-1, **(B)** RhCalnexin and **(C)** RhCytochrome c antibody.

**Additional file 5: Table S1.** Primer sequences used in PCR analysis.

**Additional file 6: Table S2.** Protein content of *Rhipicephalus haemaphysaloides* hemolymph-derived EVs identified using the MASCOT search engine

**Additional file 7: Table S3.** Protein content of *Hyalomma asiaticum* hemolymph-derived EVs identified using the MASCOT search engine

**Additional file 8: Table S4.** GO analysis of *Rhipicephalus haemaphysaloides* hemolymph-derived EVs for biological processes, cellular components and molecular functions.

**Additional file 9: Table S5.** GO analysis of *Hyalomma asiaticum* hemolymph-derived EVs for biological processes, cellular components and molecular functions.

**Additional file 10: Table S6.** KEGG pathway analysis of proteins identified from *Rhipicephalus haemaphysaloides* hemolymph-derived EVs.

**Additional file 11: Table S7.** KEGG pathway analysis of proteins identified from *Hyalomma asiaticum* hemolymph-derived EVs.

**Additional file 12: Table S8.** Protein content of *Rhipicephalus haemaphysaloides* hemolymph identified using the MASCOT search engine.

**Additional file 13: Table S9.** KEGG pathway analysis of proteins identified from *Rhipicephalus haemaphysaloides* hemolymph.

**Additional file 14: Table S10.** Unique and shared proteins identified between the tick hemolymph and EVs.

**Additional file 15: Table S11.** Host proteins identified in hemolymph EVs.

## Data Availability

All data generated or analyzed during this study are included in this published article and its additional files.
