# Supplementary material for: Proteomic analysis of extracellular vesicles from tick hemolymph and uptake of extracellular vesicles by salivary glands and ovary cells
Source: Parasit Vectors. 2023 Apr 13;16:125. doi: 10.1186/s13071-023-05753-w (PMC10100430; doi:10.1186/s13071-023-05753-w)
Supplement: Supplementary file 1 — Additional file 1: Text S1. The 4D label-free experimental procedures. [file 13071_2023_5753_MOESM1_ESM.doc]

**Additional file 1: Text S1. The 4D label-free experimental procedures**

**1. Materials and methods**

1.1 Protein extraction and digestion

SDT (4%SDS，100 mM Tris-HCl，1mM DTT，pH7.6) buffer was used for sample lysis and protein extraction. The amount of protein was quantified with the BCA Protein Assay Kit (Bio-Rad, USA). Protein digestion by trypsin was performed according to filter-aided sample preparation (FASP) procedure described by Matthias Mann. The digest peptides of each sample were desalted on C18 Cartridges (Empore™ SPE Cartridges C18 (standard density), bed I.D. 7 mm, volume 3 mL, Sigma), concentrated by vacuum centrifugation and reconstituted in 40 µL of 0.1% (v/v) formic acid.

**1.2 Filter-aided sample preparation (FASP Digestion) procedure**

200 μg of proteins for each sample were incorporated into 30 μL SDT buffer (4% SDS, 100 mM DTT, 150 mM Tris-HCl pH 8.0). The detergent, DTT and other low-molecular-weight components were removed using UA buffer (8 M Urea, 150 mM Tris-HCl pH 8.0) by repeated ultrafiltration (Microcon units, 10 kD). Then 100 μL iodoacetamide (100 mM IAA in UA buffer) was added to block reduced cysteine residues and the samples were incubated for 30 min in darkness. The filters were washed with 100 μL UA buffer three times and then 100 μL 25mM NH_4_HCO_3_ buffer twice. Finally, the protein suspensions were digested with 4 μg trypsin (Promega) in 40 μL 25mM NH_4_HCO_3_ buffer overnight at 37℃, and the resulting peptides were collected as a filtrate. The peptides of each sample were desalted on C18 Cartridges, concentrated by vacuum centrifugation and reconstituted in 40 µL of 0.1% (v/v) formic acid. The peptide content was estimated by UV light spectral density at 280 nm using an extinctions coefficient of 1.1 of 0.1% (g/l) solution that was calculated on the basis of the frequency of tryptophan and tyrosine in vertebrate proteins.

1.3 SDS-PAGE

20 µg of protein for each sample were mixed with 5× loading buffer respectively and boiled for 5 min. The proteins were separated on 12.5% SDS-PAGE gel (constant current 14 mA, 90 min). Protein bands were visualized by Coomassie Blue R-250 staining.

1.4 LC-MS/MS analysis

LC-MS/MS analysis was performed on a timsTOF Pro mass spectrometer (Bruker) that was coupled to Nanoelute (Bruker Daltonics) for 60/120/240 min. The peptides were loaded onto a reverse phase trap column (Thermo Scientific Acclaim PepMap100, 100 μm*2 cm, nanoViper C18) connected to the C18-reversed phase analytical column (Thermo Scientific Easy Column, 10 cm long, 75 μm inner diameter, 3 μm resin) in buffer A (0.1% Formic acid) and separated with a linear gradient of buffer B (84% acetonitrile and 0.1% Formic acid) at a flow rate of 300 nL/min controlled by IntelliFlow technology. The mass spectrometer was operated in positive ion mode. The mass spectrometer collected ion mobility MS spectra over a mass range of m/z 100-1700 and 1/k0 of 0.6 to 1.6, and then performed 10 cycles of PASEF MS/MS with a target intensity of 1.5k and a threshold of 2500. Active exclusion was enabled with a release time of 0.4 minutes.

1.5 Identification and quantitation of proteins

The MS raw data for each sample were combined and searched using the MaxQuant 1.5.3.17 software for identification and quantitation analysis. Related parameters and instructions are as follows:

Table Maxquant identification and quantitation indexes

| **Item** | **Value** |
| --- | --- |
| **Enzyme** | Trypsin |
| **Max Missed Cleavages** | 2 |
| **Fixed modifications** | Carbamidomethyl (C), |
| **Variable modifications** | Oxidation (M) , |
| **Main search** | 6 ppm |
| **First search** | 20 ppm |
| **MS/MS Tolerance** | 20 ppm |
| **Database** | uniprot-Argasidae  uniprot-Ixodidae  uniprot-Oryctolagus cuniculus  uniprot-Rabbit |
| **Database pattern** | Reverse |
| **Include contaminants** | True |
| **protein FDR** | ≤0.01 |
| **Peptide FDR** | ≤0.01 |
| **Peptides used for protein quantification** | Use razor and unique peptides |
| **Time window (match between runs)** | 2 min |
| **protein quantification** | LFQ. |
| **min. ratio count** | 1 |

2. Bioinformatic analysis

2.1 Cluster analysis of phosphorylated peptides

Cluster 3.0 (http://bonsai.hgc.jp/~mdehoon/software/cluster/software.htm) and Java Treeview software (http://jtreeview.sourceforge.net) were used to performing hierarchical clustering analysis. Euclidean distance algorithm for similarity measure and average linkage clustering algorithm (clustering uses the centroids of the observations) for clustering were selected when performing hierarchical clustering. A heat map was often presented as a visual aid in addition to the dendrogram.

2.2 Motif analysis

The motifs were analyzed by MeMe (http://meme-suite.org/index.htm). We extracted the amino acid sequences contain the modified site and six upstream/downstream amino acids from the modified site (13 amino acid sites in total). These sequences were used to predicted motifs in this study (parameters: width：13, occurrences:20, background: species).

2.3 Subcellular localization

CELLO (<http://cello.life.nctu.edu.tw/>) which is a multi-class SVM classification system, was used to predict protein subcellular localization.

2.4 Domain annotation

Protein sequences are searched using the InterProScan software to identify protein domain signatures from the InterPro member database Pfam.

2.5 GO annotation

The protein sequences of the selected differentially expressed proteins were locally searched using the NCBI BLAST+ client software (ncbi-blast-2.2.28+-win32.exe) and InterProScan to find homologue sequences, then gene ontology (GO) terms were mapped and sequences were annotated using the software program Blast2GO. The GO annotation results were plotted by R scripts.

2.6 KEGG annotation

Following annotation steps, the studied proteins were blasted against the online Kyoto Encyclopedia of Genes and Genomes (KEGG) database (http://geneontology.org/) to retrieve their KEGG orthology identifications and were subsequently mapped to pathways in KEGG.

2.7 Enrichment analysis

Enrichment analysis were applied based on the Fisher’ exact test, considering the whole quantified proteins as background dataset. Benjamini-Hochberg correction for multiple testing was further applied to adjust derived p-values. And only functional categories and pathways with p-values under a threshold of 0.05 were considered as significant.

2.8 Protein-protein interaction analysis

The protein–protein interaction (PPI) information of the studied proteins was retrieved from IntAct molecular interaction database (http://www.ebi.ac.uk/intact/) by their gene symbols or STRING software (http://string-db.org/). The results were downloaded in the XGMML format and imported into Cytoscape software (http://www.cytoscape.org/, version 3.2.1) to visualize and further analyze functional protein-protein interaction networks. Furthermore, the degree of each protein was calculated to evaluate the importance of the protein in the PPI network.
