# Supplementary figures and images for: Proteomic analysis of extracellular vesicles from tick hemolymph and uptake of extracellular vesicles by salivary glands and ovary cells
Source: Parasit Vectors. 2023 Apr 13;16:125. doi: 10.1186/s13071-023-05753-w (PMC10100430; doi:10.1186/s13071-023-05753-w)

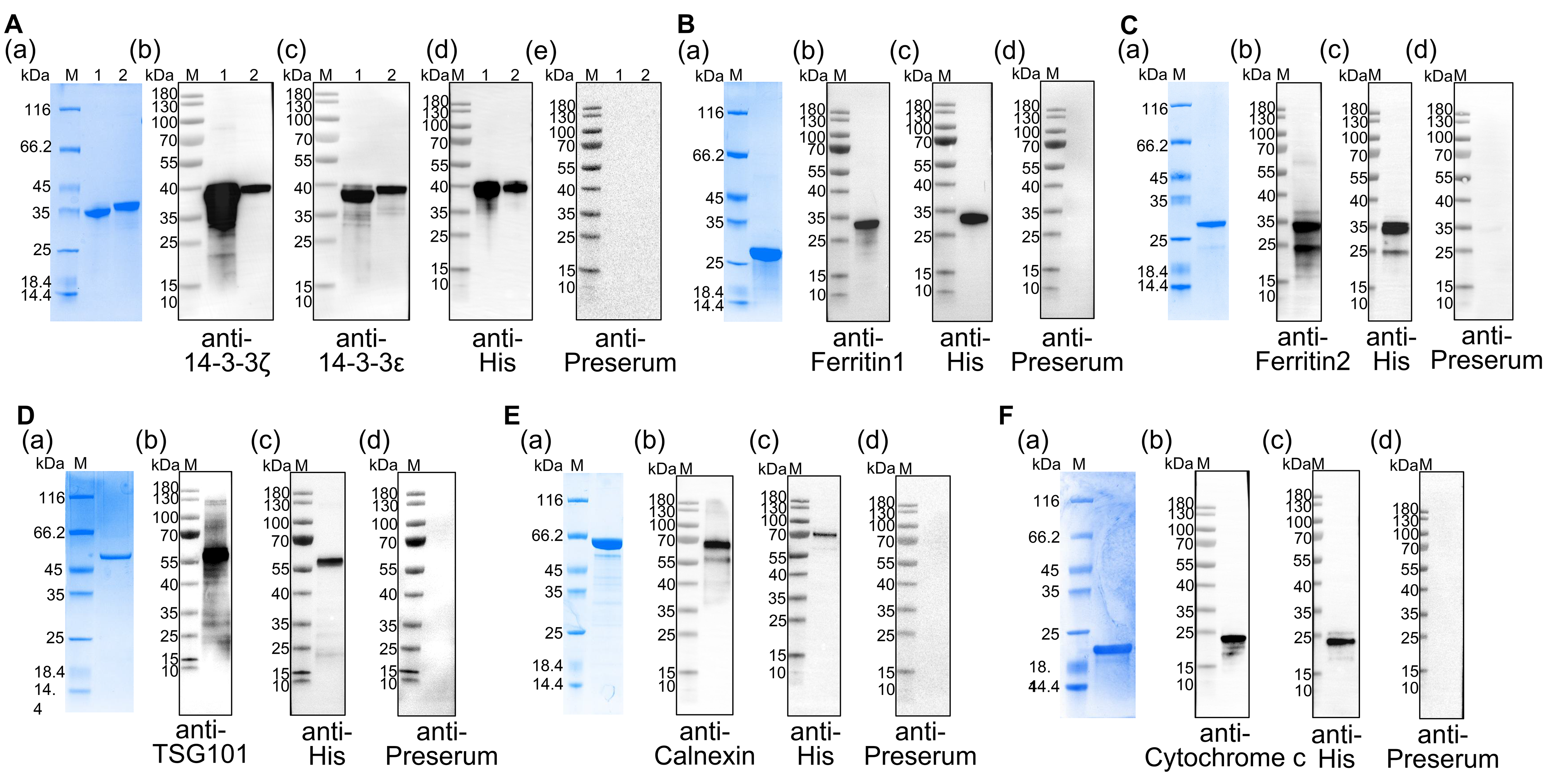

Supplement: Supplementary file 2 — Additional file 2: Figure S1. Expression and affinity purification of recombinant proteins. Recombinant proteins were purified by Ni-NTA affinity column (detected by western blot). The gel was stained with Coomassie brilliant blue G250 (CBB G250). (A) Western blot analyses of the recombinant Rh14-3-3ζ and Rh14-3-3ε protein. The (a) panel is a gel, the (b) panel is a western blot for anti-Rh14-3-3ζ, the (c) panel is a western blot for anti-Rh14-3-3ε, the (d) panel is a western blot of a 6*His-tagged fusion protein with anti-6*His tag and the (e) panel is a western blot for anti-preserum. Lane 1, recombinant Rh14-3-3ζ protein; Lane 2, recombinant Rh14-3-3ε protein. Western blot analyses of the recombinant (B) RhFerritin-1, (C) RhFerritin-2, (D) RhTSG101, (E) RhCalnexin and (F) RhCytochrome c protein. For (B)–(F), all of the (a) panels are gel, the (b) panels are western blots for anti-corresponding protein, the (c) panels are western blots of the 6*His-tagged fusion protein with anti-6*His tag and the (d) panels are western blots for anti-preserum. [file 13071_2023_5753_MOESM2_ESM.tif]

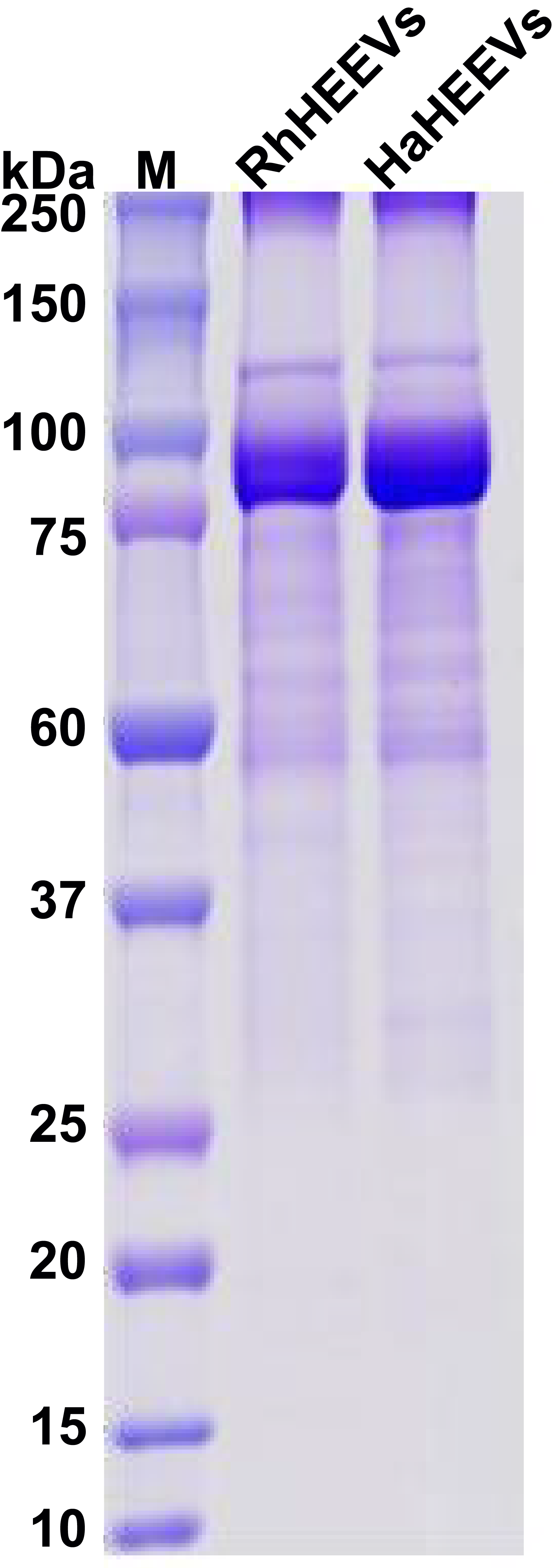

Supplement: Supplementary file 3 — Additional file 3: Figure S2. SDS-PAGE analysis of proteins from RhHEEVs and HaHEEVs. [file 13071_2023_5753_MOESM3_ESM.tif]

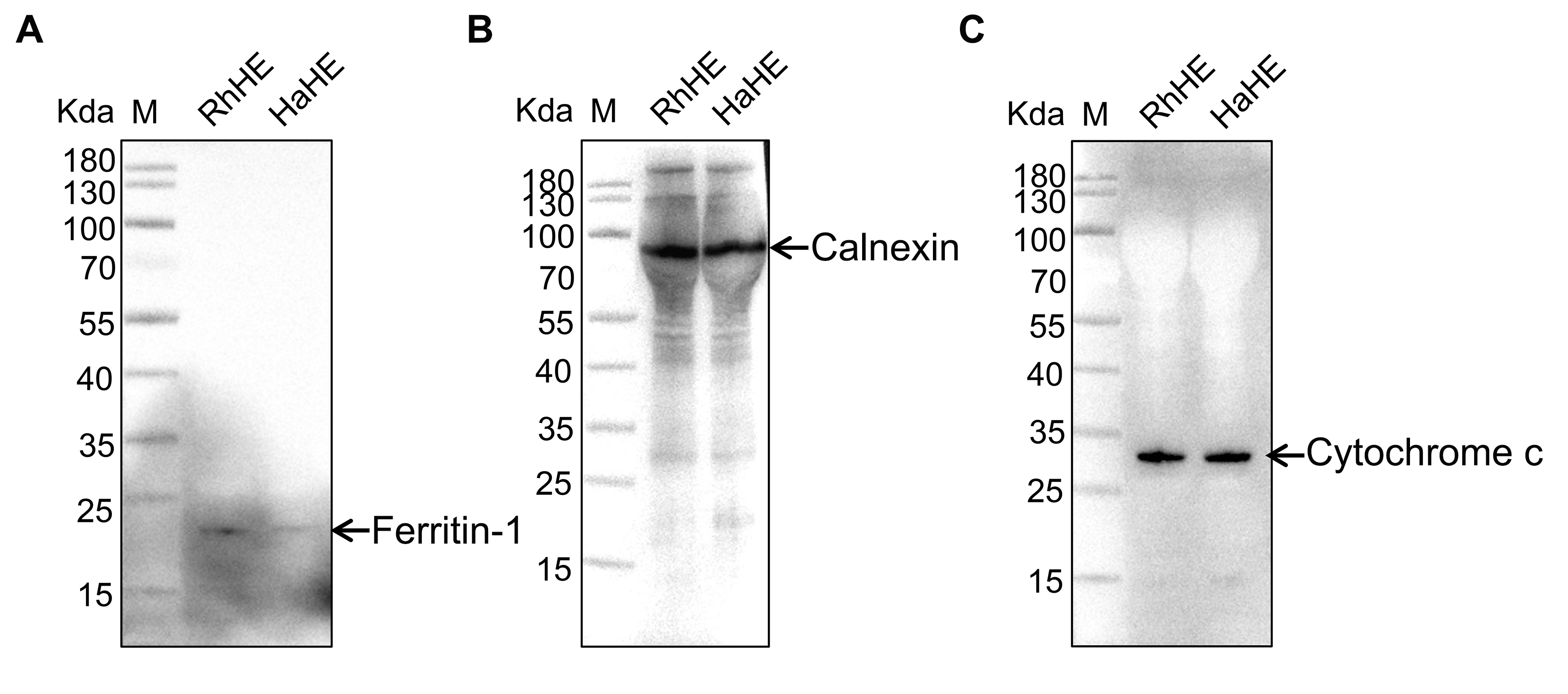

Supplement: Supplementary file 4 — Additional file 4: Figure S3. Western blot analysis of whole Rhipicephalus haemaphysaloides and Hyalomma asiaticum hemolymph lysates. A 50 μg amount of whole hemolymph lysate protein per lane was loaded. Western blot analyses of the RhHE and HaHE proteins by (A) RhFerritin-1, (B) RhCalnexin and (C) RhCytochrome c antibody. [file 13071_2023_5753_MOESM4_ESM.tif]
