# Supplementary material for: Proteomic analysis of extracellular vesicles from tick hemolymph and uptake of extracellular vesicles by salivary glands and ovary cells
Source: Parasit Vectors. 2023 Apr 13;16:125. doi: 10.1186/s13071-023-05753-w (PMC10100430; doi:10.1186/s13071-023-05753-w)
Supplement: Supplementary file 5 — Additional file 5: Table S1. Primer sequences used in PCR analysis. [file 13071_2023_5753_MOESM5_ESM.doc]

**Additional file 5: Table S1. Primer sequences used in PCR analysis.**

| **Gene name** | **Primer sequence (5′-3′)** | **Features** |
| --- | --- | --- |
| Rh14-3-3ζ | F: ATGGATAAGGACGAGTTGGTT |  |
|  | R: TCAATTGTCACCACCTTCCTG |  |
| Rh14-3-3ε | F: ATGGCCGAGAGAGAAGACA |  |
|  | R: TTACGAAACGTCTTGGTCT |  |
| RhFerritin-1 | F: ATGGCCGCTACTCAGCCCCGTC |  |
|  | R: TCAGTCTGATAGGGTCTCC |  |
| RhFerritin-2 | F: ATGCTTCGAGTCGTGCTTCTC |  |
|  | R: TTAGGTTTGCAGTTGCTG |  |
| RhCytpchrome c | F: ATGGGCGATATCCCTAAGG |  |
|  | R: CTACTTCGTGGCTTGCTCC |  |
| RhCalnexin | F: ATGTATTTAGGCTGTGCATTTT |  |
|  | R: CTAACGCACCGCTCCGTCTCC |  |
| RhTSG101 | F: ATGAAAAAGGAGCTCTTCTGCCT |  |
|  | R:CTATGGGAGTTGGGCCTTTTCT |  |
| pET30a-  Rh14-3-3ζ | F: TGGCTGATATCGGATCCATGGATAAGGACGAGTTGGTT | Protein (underlined for cleavage site) |
|  | R: GTGGTGGTGCTCGAGTCAATTGTCACCACCTTCCTG |  |
| pET30a-  Rh14-3-3ε | F: GCTGATATCGGATCCATGGCCGAGAGAGAAGACA |  |
|  | R: TGGTGGTGGTGCTCGAGTTACGAAACGTCTTGGTCT |  |
| pET30a-  RhFerritin-1 | F: ATGGCTGATATCGGATCCATGGCCGCTACTCAGCCCCG |  |
|  | R: GTGGTGGTGGTGCTCGAGTCAGTCTGATAGGGTCTCC |  |
| pET30a-  RhFerritin-2 | F: ATGGCTGATATCGGATCCATGCTTCGAGTCGTGCTTCTC |  |
|  | R: GTGGTGGTGGTGCTCGAGTTAGGTTTGCAGTTGCTG |  |
| pET30a-  RhCytpchrome c | F: TGGCTGATATCGGATCCATGGGCGATATCCCTAAGG |  |
|  | R: GTGGTGGTGCTCGAGCTACTTCGTGGCTTGCTCC |  |
| pET30a-  RhCalnexin | F: TGGCTGATATCGGATCCATGTATTTAGGCTGTGCATTTT |  |
|  | R: GTGGTGGTGCTCGAGCTAACGCACCGCTCCGTCTCC |  |
| pET30a-  TSG101 | F: GCCATGGCTGATATCGGATCCATGAAAAAGGAGCTCTTCT |  |
|  | R: GTGGTGGTGGTGGTGCTCGAGCTATGGGAGTTGGGCCTTTTCT |  |

Note: F means forward primer; R indicates reverse primer
